# 

**Published:** 1879-10

**Authors:** 


### THE CHICAGO MEDICAL Journal and Examiner

EDITORS:

WILLIAM H. BYFORD, A.M., M.D.,

JAS. NEVINS HYDE, A.M., M.D. N. S. DAVIS, M.D., LL.D.

DANIEL R. BROWER, M.D.

CHICAGO MEDICAL PRESS ASSOCIATION. AUG. 2D 1875.

CHICAGO:

*PUBLISHED BY THE CHICAGO MEDICAL PRESS ASSOCIATION.*

188 CLARK STREET.

☞Read the Notice of this Journal among Advertisements.

